# Use of topical Vitamin E in oral mucositis in patients undergoing oncology treatment. Scoping review

**DOI:** 10.4317/medoral.27790

**Published:** 2025-10-17

**Authors:** Valeria Sanmartín-Barragáns, Mencia Vázquez-Rico, Gisela Cristina Vianna Camolesi, Irene B Prado-Pena, Tamara García-Carnicero, Mario Pérez-Sayáns, Andrés Blanco-Carrión, Pilar Gándara-Vila

**Affiliations:** 1 Oral Medicine, Oral Surgery and Implantology Unit, MedOralRes Group,University of Santiago de Compostela.; 2 Health Research Institute of Santiago de Compostela (IDIS), ORALRES group, Spain; 3 University of Santiago de Compostela; 4 Materials Institute of Santiago de Compostela (iMATUS). Avenida do Mestre Mateo, 25. 15782 Santiago de Compostela, Spain

## Abstract

**Background:**

Oral mucositis (OM) refers to lesions characterized by erythema and ulceration of the oral mucosa, commonly observed in cancer patients undergoing chemotherapy (CT) and/or radiotherapy (RT). Currently, there is no specific therapy supported by robust scientific evidence for the treatment of OM-related lesions. However, the literature suggests that antioxidants such as vitamin E may help prevent oxidative cellular damage and limit tissue injury caused by free radicals, potentially reducing the severity of OM during cancer treatment. This review aims to analyze the existing literature on the use of topical vitamin E and its effects on oral mucositis lesions induced by cancer therapy.

**Material and Methods:**

This scoping review was conducted in accordance with the PRISMA-ScR guidelines. A comprehensive search was performed in Web of Science (WOS), PubMed, and Scopus databases using the keywords: "vitamin E", "oral mucositis", "chemotherapy", and "radiotherapy". Studies published between 1990 and 2025 were considered for inclusion.

**Results:**

A total of 167 articles were identified. After screening and eligibility assessment, 7 clinical trials were included in the review 4 involving pediatric populations and 3 involving adults. Six of the studies reported favorable outcomes in the vitamin E group compared to placebo, including improved healing, reduced symptom severity, and shorter duration of oral mucositis.

**Conclusions:**

Based on the studies included in this review, topical vitamin E appears to offer beneficial effects in mitigating the severity and duration of oral mucositis lesions in patients undergoing cancer treatment. However, no standardized protocol currently exists regarding dosage, mode of application (therapeutic vs. preventive), or duration of treatment. Further clinical trials are warranted to establish clear guidelines for the use of topical vitamin E in this context.

## Introduction

Oral mucositis (OM) refers to lesions characterized by erythema and ulceration of the oral mucosa, commonly observed in cancer patients receiving chemotherapy (CT) and/or radiotherapy (RT). This complication affects up to 80% of patients undergoing high-dose CT, nearly 100% of patients receiving RT for head and neck cancers, and approximately 20-40% of those treated with conventional CT regimens (1 , 2). OM is a painful and debilitating condition associated with inflammation, dysphagia, xerostomia, taste disturbances, weight loss, secondary infections, and, in some cases, halitosis and dysgeusia. These complications can significantly interfere with cancer treatment, prolong hospitalization, and negatively impact patients' quality of life (1). The development and severity of OM depend not only on treatment type, dosage, and the number of cycles but also on individual patient characteristics (3).

Several clinical scales have been developed to classify and assess OM severity, including the World Health Organization (WHO) Oral Toxicity Scale, the Children's International Mucositis Evaluation Scale (ChIMES), and the Oral Mucositis Assessment Scale (OMAS) (4).

Among the various interventions explored for OM, the efficacy of vitamin E has also been investigated (5). This compound can be used either as a standalone antioxidant-such as -tocopherol, -tocotrienol, or tocotrienol-rich fraction (TRF)-or in commercial formulations, such as vitamin E succinate (with anticancer potential) or vitamin E acetate (6 , 7). Additionally, vitamin E is present in natural products like palm oil (6).

Vitamin E is a potent antioxidant that enhances immune function (8) and has been used to manage various oral lesions by inhibiting lipid peroxidation in cell membranes (9). It prevents tissue damage, promotes cell regeneration and epithelialization of the mucosa, protects specialized cells and tissues from oxidative injury, and supports immune responses by stimulating leukocyte production (10).

Despite the high prevalence and clinical relevance of OM, there is currently no standardized therapy with strong scientific evidence supporting its use. However, existing literature suggests that antioxidants such as vitamin E may reduce oxidative stress and limit tissue damage caused by free radicals, potentially alleviating the severity of OM during cancer treatment (11 , 12).

The aim of this scoping review is to examine the existing scientific literature on the use of topical vitamin E in the management of oral mucositis in cancer patients undergoing chemotherapy and/or radiotherapy.

This review was conducted following the PRISMA extension for scoping reviews (PRISMA-ScR) guidelines (13). The research question was framed using the Concept-Context-Population (CCP) format:

What is the nature and scope of the existing literature on the use of topical vitamin E (Concept) in the treatment of oral mucositis (Population) among cancer patients undergoing chemotherapy and/or radiotherapy (Context)?.

## Material and Methods

A comprehensive literature search was conducted in July 2025 to identify relevant studies published between January 1990 and June 2025 across three major databases: PubMed, Web of Science (WOS), and Scopus. The following search strategies were employed:

1. Oral mucositis AND vitamin E.

2. Oral mucositis AND vitamin E AND radiotherapy.

3. Oral mucositis AND vitamin E AND chemotherapy.

Studies were included if they met the following criteria:

- Use of topical vitamin E or multivitamin formulations, provided the specific effect of vitamin E could be isolated.

- Published in English or Spanish.

- Included participants undergoing chemotherapy and/or radiotherapy.

- Study design limited to clinical trials.

The selection process followed the PRISMA-ScR (Preferred Reporting Items for Systematic Reviews and Meta-Analyses extension for Scoping Reviews) guidelines (13). After removing duplicates, articles were screened based on titles and abstracts. Full-text reviews were conducted for studies that fulfilled all inclusion criteria and did not meet any exclusion criteria.

Study eligibility was assessed independently by two reviewers (VSB and MVR) using a standardized, unblinded approach. In cases of disagreement, a third reviewer (PGV) was consulted to reach a consensus.

The Newcastle-Ottawa Scale was applied to assess the risk of bias in non-randomized studies, while the Jadad scale (14 , 15) was used for randomized clinical trials (RCTs).

From each included study, the following data were extracted:

- Type of oncological treatment (chemotherapy and/or radiotherapy).

- Country where the study was conducted.

- Study design (e.g., RCT, non-randomized trial, case-control study).

- Type of intervention:

Curative (intervention applied to patients with existing OM lesions).

Preventive (intervention applied before lesion onset)

- Population group (adult or pediatric), including age and gender.

- Sample size.

- Cancer type.

- Mucositis classification scale used.

- Vitamin E dose and form of administration.

- Comparative intervention (e.g., placebo, systemic vitamin E, other topical agents).

- Reported outcomes (e.g., lesion healing, symptom reduction, toxicity).

All data were extracted according to availability in the published studies and systematically organized for comparative analysis.

## Results

**Figure 1:**
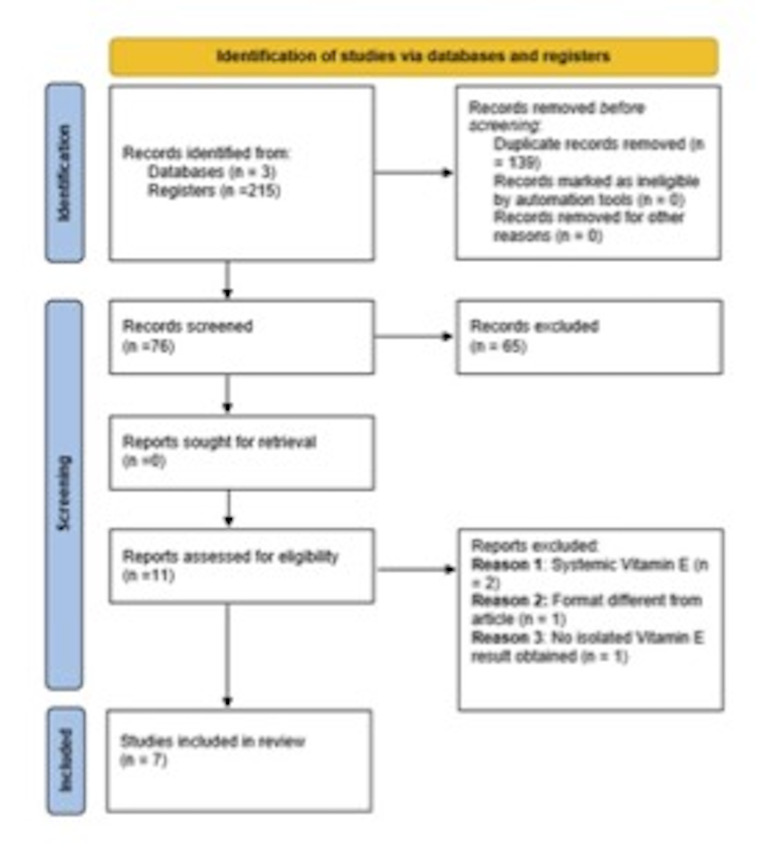
Structure of a PRISMA Flow Diagram (15).

A total of 215 articles were identified through database searches: 78 in PubMed, 23 in Web of Science, and 114 in Scopus. After removing 139 duplicates, 76 unique articles remained. Of these, 65 were excluded based on title and/or abstract for the following reasons: publication type (n=24), irrelevance to the topic (n=28), animal studies (n=5), systemic treatment focus (n=5), non-English/Spanish language (n=1), and inability to isolate the specific effect of vitamin E (n=2). As a result, 11 articles were selected for full-text review. After further evaluation, 4 were excluded (see Figure 1), leaving 7 randomized clinical trials for data extraction (16).

1

Study selection was performed independently by two reviewers (VSB and MVR), achieving an inter-reviewer agreement of 0.8 (17). Disagreements were resolved by a third reviewer (PGV). All included studies were randomized controlled trials (RCTs), and characteristics are summarized in Table 1.

Table 1

The methodological quality of studies was assessed using the Jadad scale (15). Four studies scored 2 points (4 , 10 , 18 , 19), indicating lower methodological quality, while two scored 5 points (20 - 22), reflecting a lower risk of bias.

Geographically, three studies were conducted in Asia (4 , 10 , 20), two in North America (19 , 21), one in Brazil (22), and one in Egypt (18). Only one trial (10) was conducted in a hospital setting; the rest were home-based interventions.

In terms of patient treatment, one study did not specify the cancer therapy used (10). Five studies included patients undergoing chemotherapy (4 , 18 - 21), and one included patients receiving radiotherapy (22). Most trials involved pediatric populations (4 , 10 , 18 , 21), while three focused on adults (19 , 20 , 22).

Sample sizes ranged from 16 (21) to 150 participants (10), with a total of 470 patients across all studies. Only three studies reported gender distribution (4 , 10 , 22), with more males (n=182) than females (n=94).

Regarding cancer types, two studies focused on head and neck cancer (one treated with CT (19), one with RT (22)); two included patients with acute lymphoblastic leukemia (ALL) (4 , 20); three involved acute myeloid leukemia (AML) (4 , 19 , 20); and one included non-Hodgkin lymphoma, Ewing's sarcoma, osteosarcoma, and large cell lymphoma (21). One study (19) included patients with hepatocellular carcinoma, while two did not specify the cancer type (10 , 18).

Six of the studies used the WHO Oral Toxicity Scale (4 , 10 , 18 , 19 , 21 , 22); two combined it with other scales such as OMAS and ChIMES (4), or RTOG/EORTC (22). Only one study used the NCI-CTCAE v5 as the sole scale (20).

As for comparators, four studies used placebo (19 - 22); one compared with systemic vitamin E (18); one compared with both placebo and pycnogenol (4); and one compared with chlorhexidine and honey (10).

Topical vitamin E was used as a preventive intervention in three studies (20 - 22), as a therapeutic agent in three (4 , 18 , 19), and as both in one (10).

Regarding formulation, vitamin E was administered in aqueous solution in two studies (4 , 20), in oil or oily solution in four (18 , 19 , 21 , 22), and unspecified in one (10).

Doses varied: 800 mg per day in one dose (21) or divided into two doses (19 , 20 , 22), or 200 mg administered twice (10 , 18) or three times daily (4).

All studies, except one (21), reported positive outcomes with topical vitamin E, including faster healing (4 , 18 - 20), symptom improvement (22), and reduced mucositis severity (10). No toxicity was reported in any of the included trials.

## Discussion

Oral mucositis (OM) is an inflammatory response of the oral epithelium to the cytotoxic effects of chemotherapy and radiotherapy. It is associated with pain, impaired oral intake, weight loss, and increased risk of local infections (3). As noted earlier, OM affects up to 80% of patients receiving high-dose chemotherapy and nearly all patients receiving radiotherapy for head and neck cancer (1). Despite this high incidence, there remains no widely accepted protocol for its management, potentially complicating cancer therapy and negatively affecting patient outcomes. This scoping review identified a limited body of literature on the use of topical vitamin E for OM. Of 19 full-text articles assessed, only 7 met the inclusion criteria, and just one involved patients undergoing radiotherapy (22). Six of the seven studies reported favorable outcomes for vitamin E compared to control interventions (4 , 10 , 18 - 20 , 22), including improved healing rates, symptom reduction, and lower mucositis severity. Only one study (21), which used preventive vitamin E in pediatric patients receiving doxorubicin, found no significant difference versus placebo in OM occurrence or pain relief. However, that study still reported fewer severe cases of OM and less need for parenteral nutrition in the vitamin E group. In contrast, Konuk Sener et al. found preventive vitamin E use to be significantly effective (p&lt;0.05) (10). For pediatric patients with existing OM, Khurana et al. reported healing rates of 75% in the vitamin E group versus 4.5% in the placebo group and 58.5% in the pycnogenol group (p&lt;0.001) (4). Similarly, El-Housseiny et al. found an 80% healing rate with topical vitamin E compared to 0% with systemic vitamin E (p&lt;0.001) (18). Konuk et al. also observed significantly reduced mucositis grades over time compared to chlorhexidine and honey (10). Pediatric patients represented 67.7% of the total population in this review (n=318) (4 , 10 , 18 , 21), and most studies in this group showed positive outcomes, except for Sung et al. (21). The literature suggests that younger patients-especially those with hematologic malignancies-may be more prone to OM due to intense myelosuppression and high mitotic rates (23 , 24). However, the lack of benefit observed by Sung et al. cannot be solely attributed to the type of malignancy or chemotherapeutic agents, as other studies using doxorubicin or methotrexate (MTX) also reported favorable outcomes. The heterogeneity in chemotherapeutic regimens complicates interpretation. While Sung et al. used doxorubicin (with some prior MTX exposure), other studies used cyclophosphamide, cisplatin, cytarabine, or combinations with MTX and cyclosporine (19 , 20). Notably, Wadleigh et al. also used doxorubicin and observed significantly better healing in the vitamin E group compared to placebo (6/9 vs. 1/9 patients) (19). A lack of consistency was observed in the use of mucositis grading scales. While the WHO scale was used in 6 of the 7 studies, some combined it with ChIMES, OMAS, or RTOG/EORTC, as supported by Khurana et al. (4). Using multiple scales may improve the assessment of different parameters, though it complicates comparisons across studies. Authors such as López-Castaño et al. and Docimo et al. have emphasized the need for standardized tools, particularly in pediatric populations (25 , 26). Only three studies reported gender, all showing male predominance (4 , 10 , 22). This may reflect higher incidence rates of the included cancer types-particularly leukemia, non-Hodgkin lymphoma, and head and neck cancer-in male patients (27 - 30). Additionally, alcohol and tobacco use, major risk factors for head and neck cancer, are more prevalent in males, potentially explaining this disparity (30 , 31). Substantial variability was observed in dosage, posology, and treatment duration. Adult studies used a consistent daily dose of 800 mg, while pediatric doses were generally 200 mg, administered two or three times daily. Treatment durations ranged from 5 days to several weeks or throughout the cancer therapy course (10 , 18 - 22). Only two studies justified their dosing based on previous literature (21 , 22), while others appear to have adopted similar regimens without a clear rationale (4 , 10 , 18). The lack of a standardized vitamin E protocol for OM management contrasts with the well-established systemic vitamin E protocols used for osteoradionecrosis (e.g., 1000 mg vitamin E+800 mg pentoxifylline per day) (32). Topical administration appears more effective and safer than systemic use. El-Housseiny et al. demonstrated superior outcomes with topical versus systemic vitamin E (18). Ghoreishi et al. also found systemic vitamin E to be ineffective, possibly due to interference with chemotherapy (33). In contrast, topical application showed no adverse effects and did not compromise oncological treatment (19 , 22). Notably, there is a lack of studies on the use of topical vitamin E for OM in patients with solid tumors, such as breast cancer. OM has been reported in up to 76.5% of breast cancer patients receiving chemotherapy (34), and other interventions, including dexamethasone (35 , 36) and herbal products like Plantago ovata (37), have been studied in this context. However, no studies on topical vitamin E were identified in this patient group. Finally, the variability in OM grading systems remains a key barrier to comparing study outcomes. Given its simplicity and widespread use, the WHO scale may serve as a universal baseline tool, with supplemental scales added to capture additional clinical information, as suggested by Khurana et al. and supported by previous literature (4 , 23).

## Conclusions

Based on the studies included in this review, topical vitamin E appears to offer beneficial effects in mitigating the severity and duration of oral mucositis lesions in patients undergoing cancer treatment. However, no standardized protocol currently exists regarding dosage, mode of application (therapeutic vs. preventive), or duration of treatment. Further clinical trials are warranted to establish clear guidelines for the use of topical vitamin E in this context.

## Figures and Tables

**Table 1:** Table Data table of included studies.

	Study	Country	Type of Study	Type of Intervention	Population group /Age	Gender	Sample Size	Type of Cancer	Scale Used	Dose/Treatment Administered	Intervention	Comparative Intervention	Results
CMT*	El-Housseiny et al. 2007	Egypt	Randomized trial	Curative	Pediatrics Under 12 years	-	80	-	WHO Scale	-	100 mg Vitamin E capsule twice daily	Intake of 100 mg Vitamin E capsule twice daily	80% total cure of OM in 5 days (p<0.001). No cure observed in placebo group
CMT	Khurana et al. 2013	India	Randomized trial	Curative	Pediatrics 6-15 years	57 M*, 15 F*	72	ALL*, AML*, NHL*	WHO Scale, OMAS, ChIMES	-	200 mg of Vitamin E in aqueous solution daily, 3 times/day for 1 week	Pycnogenol in aqueous solution 1 mg/kg per day, 3 times/day for 1 week. Glycerin in aqueous solution	Vitamin E: 75% complete cure.Pycnogenol: 58.3% complete cure / 4.2% no improvement.Control: 4.2% cure / 83.3% no improvement
CMT	Solduzian et al. 2021	Iran	Double-blind, placebo-controlled randomized trial	Preventive	Adults	-	80	ALL, AML	NCICTCEA v5 Scale	16 doses: Busulfan 0.8 mg/kg IV, 2 doses: cyclophosphamide 60 mg/kg	400 IU of Vitamin E in aqueous solution twice daily (total of 14 days, starting with chemotherapy and ending 7 days post-transfusion)	Placebo twice daily (total of 14 days)	Reduces average healing time by 3 days in Vitamin E group. Placebo group average healing time is 4 days. P=0.02. Does not reduce OM incidence. No decrease in OM severity, p=0.35
CMT	Sung et al. 2007	Canada	Double-blind, placebo-controlled randomized trial	Preventive	Pediatrics, Average age 12.7 years	-	16	Ewing's Sarcoma (most common)	WHO Scale	Doxorubicin, methotrexate in some cycles	800 mg of Vitamin E in oil once daily for 2 weeks	Placebo once daily for 2 weeks	No significant differences in mean OM values between Vitamin E group (0.2) and placebo (0.3). The proportion of days with mucositis in both groups is 26%. Presence of severe mucositis: Vitamin E (1.5%), placebo (2.1%)
CMT	Wadleigh et al. 1992	USA	Double-blind, placebo-controlled randomized trial	Curative	Adults, Average age 61.3 years	-	18	HCN *, AML, EP*, HCC*	WHO Scale	Cisplatin, Doxorubicin, 5-fluorouracil, Cytarabine	Vitamin E oil 400 mg twice daily for 5 days	Placebo: soybean and coconut oil	6/9 patients completely cured in 5 days (average healing time 3 days). Only 1/9 patients cured in 5 days. P=0.025
There is no record	Konuk Sener et al. 2019	Turkey	Randomized controlled trial with parallel group design	Curative/Preventive	Pediatrics, Minimum age 2 years	77 M, 73 F	150 (75 with OM, 75 without)	-	WHO Scale	-	100 IU of topical Vitamin E twice daily	Honey 1-1.5 g per kg daily, twice daily. Chlorhexidine twice daily	Group 1 had higher mucositis indices than Group 2 (p<0.05). Group 2 had lower OM values than Group 3 (p<0.05). Group 4 had higher OM values on days 9-12-15 compared to Groups 5 and 6 (p<0.05). OM in Group 4 higher than initial values (p<0.05)
RTP*	Ferreira et al. 2004	Brazil	Double-blind randomized trial	Preventive	Average age 55.4 years	48 M, 6 F	54 (28 Vitamin E, 26 Placebo)	OC*, ORP*	WHO Scale, RTOG/EORTC	50-60 Gy/5-6 weeks	400 mg Vitamin E capsule in oil: dissolution in mouth for 5 minutes twice daily during radiotherapy	500 mg placebo capsule in oil twice daily during radiotherapy	21.6% of symptomatic patients in the Vitamin E group and 33.5% in the placebo group. P=0.038.Vitamin E reduces risk by 36%. Vitamin E decreases pain and food restriction from Grade 2 to 3 during radiotherapy, P=0.001

*CMT: Chemotherapy. RTP: Radiotherapy. M: Male. F: Female. ALL: Acute lymphocytuc leukemia. AML: Acute myeloid leukemia. NHL: Non-Hodgkin´s lymphoma. HCN: Head and neck. EP: Esophagus. ORP: Oropharynx. OC: Oral cavity. HCC: Hepatocellular carcinoma*

## Data Availability

Delared none.
